# Maternal gene *Ooep* may participate in homologous recombination-mediated DNA double-strand break repair in mouse oocytes

**DOI:** 10.24272/j.issn.2095-8137.2018.067

**Published:** 2018-06-15

**Authors:** Da-Jian He, Lin Wang, Zhi-Bi Zhang, Kun Guo, Jing-Zheng Li, Xie-Chao He, Qing-Hua Cui, Ping Zheng

**Affiliations:** 1 Yunnan Key Laboratory of Animal Reproduction, Kunming Institute of Zoology, Chinese Academy of Sciences, Kunming Yunnan 650223, China; 2 Laboratory of Biochemistry and Molecular Biology, School of Life Sciences, Yunnan University, Kunming Yunnan 650091, China; 3 Kunming College of Life Science, University of Chinese Academy of Sciences, Kunming Yunnan 650204, China

**Keywords:** *Ooep*, Homologous recombination, DNA double-strand break repair, ATM, RAD51

## Abstract

DNA damage in oocytes can cause infertility and birth defects. DNA double-strand breaks (DSBs) are highly deleterious and can substantially impair genome integrity. Homologous recombination (HR)-mediated DNA DSB repair plays dominant roles in safeguarding oocyte quantity and quality. However, little is known regarding the key players of the HR repair pathway in oocytes. Here, we identified oocyte-specific gene *Ooep* as a novel key component of the HR repair pathway in mouse oocytes. OOEP was required for efficient ataxia telangiectasia mutated (ATM) kinase activation and Rad51 recombinase (RAD51) focal accumulation at DNA DSBs. *Ooep* null oocytes were defective in DNA DSB repair and prone to apoptosis upon exogenous DNA damage insults. Moreover, *Ooep* null oocytes exhibited delayed meiotic maturation. Therefore, OOEP played roles in preserving oocyte quantity and quality by maintaining genome stability. *Ooep* expression decreased with the advance of maternal age, suggesting its involvement in maternal aging.

## INTRODUCTION

During the long period of meiotic arrest, oocytes are exposed to endogenous and exogenous genotoxic insults and tend to accumulate DNA damage. DNA damage can occur as single-stranded breaks (SSBs) or double-strand breaks (DSBs), the latter of which are highly deleterious and can substantially impair genomic integrity and cell viability. Two major pathways are involved in DNA DSB repair, non-homologous end joining (NHEJ)- and homologous recombination (HR)-mediated repair. In oocytes, defection of the error-free HR pathway can cause a decline in oocyte quantity and quality in natural and pathological reproductive aging in both mice and humans (Day et al., 2015; Perry et al., 2013; Titus et al., 2013; Wang et al., 2014). Therefore, HR-based DNA DSB repair in oocytes plays a key role in regulating female reproductive performance. This repair has been intensively studied in somatic cells, with many players identified (Adamson et al., 2012); however, little information is available regarding the components of the HR repair pathway in oocytes. Identifying essential participants will shed light on understanding oocyte and ovarian aging.

Oocyte-expressed protein (*Ooep*) is an oocyte-specific maternal-effect gene. The OOEP protein localizes at the subcortical region of an oocyte and interacts with four other maternal proteins, including MATER, FILIA, PADI6, and TLE6, to form a subcortical maternal complex (SCMC) in mouse and human oocytes (Li et al., 2008; Zhu et al., 2015). Depletion of OOEP or its interaction proteins can lead to embryo arrest at the 2-cell stage with uncharacterized molecular mechanisms in mice (Li et al., 2008; Tong et al., 2000; Yu et al., 2014; Yurttas et al., 2008; Zheng & Dean, 2009). We recently utilized mouse embryonic stem cells as a model to explore the function of FILIA and uncovered its critical roles in safeguarding the genomic stability of pluripotent stem cells (Zhao et al., 2015). This finding prompted our hypothesis that OOEP may be involved in regulating genome stability in oocytes. Thus, in this study, we carefully examined the function of OOEP in DNA DSB repair in oocytes.

## MATERIALS AND METHODS

### Animals

The mouse line with targeted mutation of *Ooep* (background C57BL/B6×Sv129) was kindly provided by Dr. Jurrien Dean from the National Institutes of Health, USA. These *Ooep^tm/tm^* female mice do not express the OOEP protein in oocytes (Li et al., 2008). Mice were maintained in specific pathogen-free conditions. All experimental procedures and animal care were performed according to the protocols approved by the Ethics Committee of the Kunming Institute of Zoology, Chinese Academy of Sciences.

### DNA damage treatment of oocytes and ovaries

Germinal vesicle (GV)-stage oocytes were cultured according to standard procedures (Marangos & Carroll, 2012). Ovaries were collected from newborn mice at postnatal day 5 (P5) and cultured according to standard protocols (Gonfloni et al., 2009).

### Antibodies

Rabbit anti-OOEP serum was raised against the 1–19 amino acids of the OOEP protein (Li et al., 2008), and antibody specificity was verified by utilizing *Ooep* null oocytes. Other primary and secondary antibodies were obtained commercially, with relevant information shown in Table 1.

**Table 1:** Information on primary and secondary antibodies for immunofluorescence staining (IF) and immunoblotting (IB)

Antibody	Company	Host	Catalog number	Dilution
**Primary**				
γ-H_2_AX	Cell signaling, USA	Rabbit	9718S	1:200 (IF) 1:800 (IB)
p-ATM(Ser1981)	Novus biologicals, USA	Mouse	NB100-306	1:200 (IF) 1:800 (IB)
RAD51	Abnova, China	Mouse	H00005888-B01P	1:200 (IF) 1:500 (IB)
DDX4	Abcam, USA	Rabbit	AB13840	1:200 (IF)
Beta-ACTIN	Zhongshan, China	Mouse	TA09	1:5000 (IB)
GFP	Abcam, USA	Chicken	AB13970	1:2000 (IB)
**Secondary**				
Alexa Fluor 488 Donkey anti-Rabbit IgG	Invitrogen, USA		A-21206	1:500 (IF)
Alexa Fluor 555 Donkey anti Rabbit IgG	Invitrogen, USA		A-31572	1:500 (IF)
Alexa Fluor 647 Goat anti Rabbit IgG	Invitrogen, USA		A-21244	1:500 (IF)
Alexa Fluor 488 Goat anti Mouse IgG	Invitrogen, USA		A-11029	1:500 (IF)
Alexa Fluor 555 Donkey anti Mouse IgG	Invitrogen, USA		A-31570	1:500 (IF)
Alexa Fluor 488 Goat anti Chicken IgG	Invitrogen, USA		A-11039	1:500 (IF)

### Immunofluorescence staining and foci intensity quantification

Immunofluorescence staining of oocytes and ovaries was performed according to standard procedures (Guo et al., 2016; Xiong et al., 2008). DNA was labeled with 4${}^{\prime }$, 6-diamidino-2-phenylindole (DAPI, Sigma, Germany). Images were analyzed using FV10-ASW 2.1 viewer software. The intensities of the foci were quantified using Image J software.

### Comet assay

Alkaline comet assay was performed according to standard procedures (Berthelot-Ricou et al., 2011; Tice et al., 2000). Comets were analyzed using CASP comet assay analysis software (Andor Technology, UK). Three independent repeats were performed.

### Detection of apoptosis by TUNEL staining

Frozen ovarian sections were permeabilized with 0.1% Triton X-100 in phosphate-buffered saline (PBS), followed by incubation with the TUNEL reaction solution from the *In-Situ* Cell Death Detection Kit, Fluorescein (Roche Diagnostics, USA). All staining procedures were performed according to the manufacturer’s instructions. DNA was labeled with DAPI.

### Hematoxylin and eosin (HE) staining and primordial and primary follicle counting

Ovarian sections were incubated with the HE reaction solution (BOSTER, USA) at room temperature (RT). All staining procedures were performed according to the manufacturer’s instructions. Oocytes residing in primordial and primary follicles were counted, as described previously (Myers et al., 2004; Skaznik-Wikiel et al., 2007).

### Construction of OOEP-GFP expression plasmid

The protein-coding region of *Ooep* was amplified by polymerase chain reaction (PCR) and inserted into pcDNA3.1/CT-GFP-TOPO (Invitrogen, USA). Integrity was confirmed by DNA sequencing.

### *In vitro* transcription and mRNA microinjection of GV oocytes

*Ooep*-*Gfp* expressing plasmids were linearized with ScaI (New England Biolabs, USA). The mRNA was synthesized using an *in vitro* transcription kit (mMessage mMachine T7 kit, Ambion, USA), and purified with a RNeasy MinElute Cleanup Kit (Qiagen, Germany). The mRNA was then dissolved in nuclease-free water and stored at −80 ${}^{\circ }$C. We microinjected 500 ng/μL of mRNA in injection buffer (10 mmol/L Tris-HCl (pH 7.5) and 0.1 mmol/L EDTA) into the cytoplasm of *Ooep*-/- GV oocytes.

### Single-oocyte cDNA amplification and quantitative RT-PCR

Briefly, cDNA was prepared from a single GV oocyte and amplified by 20 cycles of PCR according to published protocols (Tang et al., 2010). Glyceraldehyde 3-phosphate dehydrogenase (*Gapdh*) was used as the housekeeping control. Primers for amplification of *Gapdh* and *Ooep* included: *Ooep* forward: 5${}^{\prime }$-GTCATAGGCACAGACCAAGCG-3${}^{\prime }$, *Ooep* reverse: 5${}^{\prime }$-GGCCGCCATGTTCAAGAGAAT-3${}^{\prime }$; *Gapdh* forward: 5${}^{\prime }$-TTGAGGTCAATGAAGGGGTC-3${}^{\prime }$, *Gapdh* reverse: 5${}^{\prime }$-TCG TCCCGTAGACAAAATGG-3${}^{\prime }$. GraphPad Prism 5 software was used for statistical analysis.

### Statistical analyses

Quantitative analyses were based on at least three independent repeats and results were represented as means±*SEM*. Data were first tested by the homogeneity of variance test. For non-normal distribution, the data were subjected to nonparametric tests. Otherwise, the data were analyzed by *t*-tests. We considered *P*<0.05 as statistically significant.

## RESULTS

### *Ooep* participates in DNA double-strand break repair in mouse oocytes

**Figure 1:**
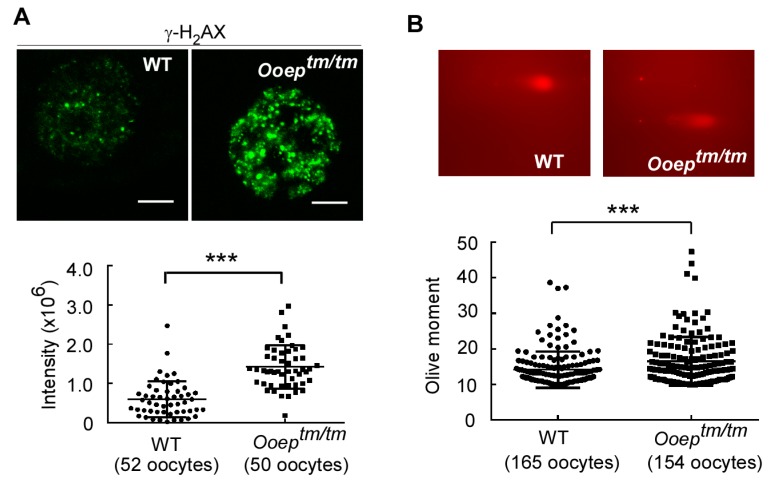
*Ooep* depletion causes DNA damage in mouse oocytes

Upon DNA damage, histone H2AX is phosphorylated at Ser139 (γ-H_2_AX) and recruited to the damaged sites to form visible foci under confocal microscopy (Rogakou et al., 1998). γ-H_2_AX foci formation is generally considered as a marker of DNA damage. In the fully-grown GV oocytes, depletion of OOEP caused a significant increase in γ-H_2_AX foci intensity compared to wild-type oocytes (Figure 1A), suggesting more endogenous DNA damage in *Ooep^tm/tm^* oocytes. To validate this observation, we performed comet assay, an unambiguous method that measures the extent of DNA damage on a single cell basis (Berthelot-Ricou et al., 2011; Tice et al., 2000). The GV oocytes from *Ooep^tm/tm^* females displayed significantly longer comet tails than those from the wild-type counterparts (Figure 1B), confirming that *Ooep^tm/tm^* oocytes contained more endogenous DNA damage.

A: Immunostaining revealed higher levels of γ-H_2_AX foci in *Ooep^tm/tm^* GV oocytes than in wild-type (WT) counterparts. Quantification of the γ-H_2_AX foci intensity is shown in the lower panel. B: Comet assay confirmed that *Ooep^tm/tm^* GV oocytes showed greater DNA damage. Data are presented as mean±*SEM*. Scale bars: 10 μm, ***: *P*<0.001.

**Figure 2:**
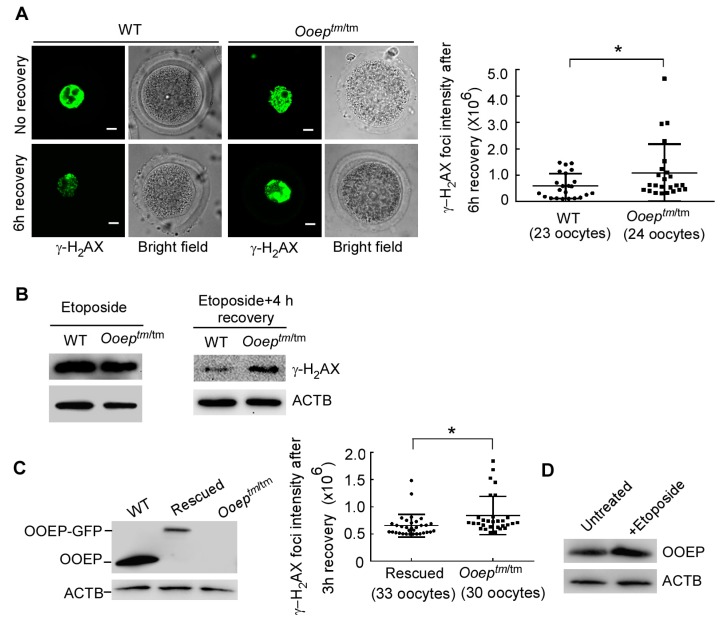
*Ooep* participates in DNA double-strand break repair in mouse oocytes

The accumulation of DNA damage in *Ooep^tm/tm^* oocytes suggested inefficiency in DNA damage repair. To test this hypothesis, we treated wild-type and mutant GV oocytes with 50 μg/mL of etoposide, a topoisomerase II inhibitor, to induce DNA DSBs (Marangos & Carroll, 2012; Nagy & Soutoglou, 2009), and compared the dynamics of γ-H_2_AX resolution. Immediately after treatment, wild-type and mutant oocytes had comparable γ-H_2_AX levels, as measured by immunostaining (Figure 2A) and immunoblotting analyses (Figure 2B). Following several hours of DNA repair recovery, γ-H_2_AX was significantly resolved in the wild-type oocytes, reflecting efficient DNA damage repair. In sharp contrast, γ-H_2_AX remained at a higher level in the *Ooep^tm/tm^* oocytes than in the wild-type oocytes, indicating compromised DNA damage repair (Figure 2A, B). To further validate the role of OOEP in DNA damage repair, we performed a rescue experiment by micro-injecting *Gfp* (green fluorescent protein)-tagged *Ooep* mRNA into the *Ooep^tm/tm^* oocytes. Consistently, the OOEP-complemented oocytes resolved γ-H_2_AX more efficiently than the *Ooep^tm/tm^* oocytes after etoposide treatment and recovery (Figure 2C). These data together support the notion that maternal protein OOEP is necessary for efficient DNA DSB repair in oocytes. In line with this role, the protein expression of OOEP was slightly induced by etoposide treatment in GV oocytes (Figure 2D).

A: *Ooep^tm/tm^* and WT oocytes were subjected to etoposide treatment and recovery. Immediately after etoposide treatment (no recovery), *Ooep^tm/tm^* and WT oocytes showed a similar level of γ-H_2_AX. After 6-h recovery, *Ooep^tm/tm^* oocytes retained more γ-H_2_AX foci than WT oocytes. B: Etoposide treatment induced a similar increase in γ-H_2_AX in both WT and *Ooep^tm/tm^* oocytes. γ-H_2_AX was better resolved in WT but not *Ooep^tm/tm^* oocytes after 4-h recovery. Each sample contained 80 oocytes. C: OOEP-GFP was re-expressed in *Ooep^tm/tm^* oocytes (left panel). After 3-h recovery from the same etoposide treatment, OOEP-rescued oocytes had a lower level of γ-H_2_AX than *Ooep^tm/tm^* oocytes (right panel). D: Immunoblotting analysis showed that OOEP protein level were upregulated by etoposide treatment. Each sample contained 20 oocytes. Data are presented as mean±*SEM*. Scale bars: 10 μm. *: *P*<0.05.

### *Ooep* may be involved in HR-mediated DNA DSB repair

**Figure 3:**
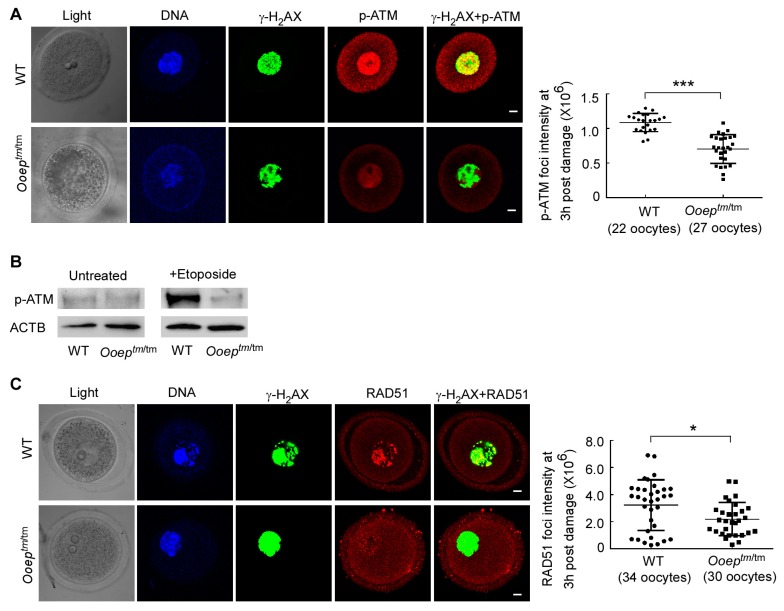
OOEP is required for ATM activation and RAD51 recruitment to DNA damage sites

In oocytes, DNA DSB is predominantly repaired via the HR-mediated pathway, in which ataxia telangiectasia mutated (ATM) kinase plays a central orchestration role (Oktay et al., 2015; Titus et al., 2013). ATM activation by DSBs initiates robust checkpoint signaling as well as DSB repair processes in somatic cells (Behrens et al., 2014; Jackson & Bartek, 2009) and germ cells (Di Giacomo et al., 2005; Oktay et al., 2015). In mouse GV oocytes, ATM can be activated by certain levels of etoposide-induced DNA DSBs. We therefore treated the oocytes with 50 μg/mL of etoposide for 3 h, as per previous study (Marangos & Carroll, 2012), and examined the effect of OOEP on ATM activation by phosphorylation at serine 1981 residue (p-ATM) (Lavin & Kozlov, 2007). Co-immunostaining analysis showed that fewer p-ATM foci were formed and co-localized with γ-H_2_AX foci in the *Ooep^tm/tm^* oocytes than in the wild-type oocytes, despite γ-H_2_AX level being comparable (Figure 3A). Immunoblotting analysis of p-ATM further validated this observation. Under normal conditions, very little p-ATM was detected in either wild-type or mutant oocytes. However, higher levels of p-ATM were induced in wild-type oocytes compared with mutant oocytes following etoposide treatment (Figure 3B). These results suggest that OOEP regulates ATM activation upon DNA DSBs.

A: *Ooep^tm/tm^* and WT oocytes were subjected to the same etoposide treatment. Immunofluorescence staining showed that at 3-h post damage, *Ooep^tm/tm^* oocytes contained a lower level of active ATM (p-ATM) compared with WT counterparts. B: Immunoblotting detection of p-ATM level under untreated and etoposide-treated conditions. In untreated conditions, rare p-ATM was detected in WT and *Ooep^tm/tm^* oocytes. At 3 h after etoposide treatment, higher level of p-ATM were detected in WT but not *Ooep^tm/tm^* oocytes. Each sample contained 80 oocytes. C: Immunofluorescence staining showed that at 3-h post etoposide treatment, *Ooep^tm/tm^* oocytes had a lower level of RAD51 than WT controls. Data are presented as mean±*SEM*. Scale bars: 10 μm. ***: *P*<0.001, *: *P*<0.05.

RAD51 is an essential recombinase downstream of ATM in the HR pathway (Oktay et al., 2015, Perez et al., 2007). After DNA DSBs, the RAD51 protein is recruited and binds to resected single-strand DNA to form DNA-protein filaments (RAD51 foci), which facilitate the searching and pairing of DNA homologues during recombination (Jasin & Rothstein, 2013). Upon etoposide treatment, RAD51 foci were formed at damage sites labeled with γ-H_2_AX in wild-type oocytes. However, fewer RAD51 foci were detected in *Ooep^tm/tm^* oocytes (Figure 3C). Thus, the absence of OOEP in oocytes impaired RAD51 focal accumulation at DNA damage sites. Taken together, these data demonstrate that OOEP may function in the HR-mediated DNA DSB repair pathway by regulating ATM activation and RAD51 recruitment to DSB sites in mouse oocytes.

### *Ooep* protects oocytes from DNA DSB-induced apoptosis and meiosis delay

**Figure 4:**
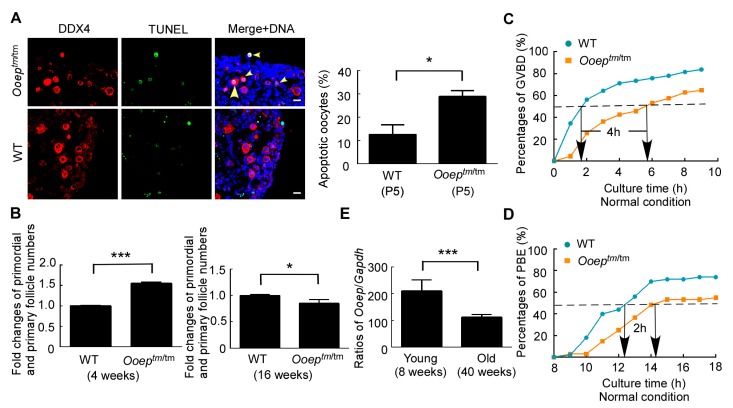
*Ooep* protects oocytes from DNA DSB-induced apoptosis and meiosis delay

Persistent DNA DSBs can evoke *p63*-mediated apoptosis of oocytes in primordial and primary follicles (Gonfloni et al., 2009; Suh et al., 2006). OOEP is necessary for HR-mediated DNA DSB repair in oocytes, suggesting that *Ooep^tm/tm^* oocytes might be sensitive to exogenous or endogenous DNA damage insults and prone to apoptosis. To test this hypothesis, we compared the apoptosis susceptibility of oocytes to exogenous DNA damage insults between wild-type and *Ooep^tm/tm^* females at the early postnatal stage. Five-day-old (P5) mouse ovaries, which contained mostly primordial follicles, were collected and cultured for 12 h with various doses of DNA cross-linking agent cisplatin (Gonfloni et al., 2009). At the concentration of 3 mg/L, cisplatin was able to induce mild apoptosis (∼13.5%) in wild-type ovaries and was therefore utilized in the following studies. After *in vitro* treatment, ovarian sections from *Ooep^tm/tm^* and wild-type females were counterstained with DDX4 (a germ cell specific marker) and TUNEL. The percentages of apoptotic oocytes (DDX4^+^TUNEL^+^) were significantly higher in *Ooep^tm/tm^* infants than in wild-type counterparts (Figure 4A), suggesting that *Ooep^tm/tm^* oocytes were more sensitive than wild-type oocytes to exogenous DNA damage insults due to compromised HR repair. To understand the *in vivo* effect of OOEP depletion under the physiological conditions, we collected ovaries from 4-week-old and 16-week-old wild-type and *Ooep^tm/tm^* females and compared the number of oocytes within primordial and primary follicles, which express P63 and are subject to DNA damage-induced atresia (Suh et al., 2006). Intriguingly, at 4-week old, the numbers of oocytes in primordial and primary follicles were significantly higher in *Ooep^tm/tm^* females than in wild-type females. This probably reflected the developmental halt caused by the accumulated endogenous DNA damage in part of the oocytes. In contrast, at 16-week old, the numbers of oocytes in primordial and primary follicles were statistically lower in *Ooep^tm/tm^* females than in wild-type females (Figure 4B). Together, these results support that OOEP protects oocytes from endogenous or exogenous DNA DSB-induced apoptosis and preserves oocyte quantity under DNA damage insults.

A: Postnatal day 5 (P5) ovaries from WT and *Ooep^tm/tm^* females were subjected to the same cisplatin treatment. TUNEL examination showed that the numbers of apoptotic germ cells (DDX4^+^TUNEL^+^, arrowheads) were higher in *Ooep^tm/tm^* ovaries than in WT ovaries. B: HE staining revealed that ovaries from 4-week-old *Ooep^tm/tm^* females contained more primordial and primary follicles than those from WT females (left panel). At 16-week old, *Ooep^tm/tm^* ovaries contained less primordial and primary follicles than WT ovaries (right panel). Ten ovarian sections were examined for each animal and five females were used at each stage. C: The germinal vesicle breakdown (GVBD) of *Ooep^tm/tm^* oocytes was delayed compared to the WT oocytes under normal conditions. There was a 4-h difference in the timing of 50%-GVBD between the two samples. More than 80 oocytes were used in each sample. D: Kinetics of the first polar body extrusion (PBE) in WT and *Ooep^tm/tm^* GV oocytes under normal conditions. *Ooep^tm/tm^* oocytes displayed 2-h delay in reaching 50%-PBE. More than 50 oocytes were used in each sample. E: mRNA expression of *Ooep* declined with maternal aging in GV oocytes. Fourteen single GV oocytes from two females were examined at each stage. Data are presented as mean±*SEM*. *: *P*<0.05, **: *P*<0.01, ***: *P*<0.001.

DNA damage in fully grown oocytes compromises the completion of meiosis by inducing a delay in meiosis resumption (germinal vesicle breakdown, GVBD) (Marangos & Carroll, 2012) and the activation of spindle assembly checkpoint (SAC) at metaphase I (Collins et al., 2015; Marangos et al., 2015). We investigated the influence of OOEP loss on the kinetics of GVBD and the first polar body extrusion (PBE) during oocyte *in vitro* maturation. Under normal conditions, the *Ooep^tm/tm^* oocytes showed a 4-h delay in reaching 50%-GVBD compared with wild-type oocytes. In addition, ∼20% less oocytes completed GVBD in the absence of OOEP compared with the wild-type counterparts at the end of culture (Figure 4C). Likewise, the *Ooep^tm/tm^* oocytes displayed a 2-h delay in reaching 50%-PBE. At the end of culture, 20% less oocytes completed PBE compared with the wild-type counterparts (Figure 4D). Thus, OOEP was essential for efficient meiosis resumption and completion by preserving oocyte genomic integrity. Decline in DNA DSB repair competence is considered a major factor contributing to oocyte and ovarian aging at advanced maternal age (Oktay et al., 2014, 2015; Titus et al., 2013; Xu et al., 2015). We examined if *Ooep* expression declined with maternal aging. mRNA expression levels of the *Ooep* gene measured in single GV oocytes of old females (40-week old) were lower than those from young females (8-week old) (Figure 4E). Thus, *Ooep* expression exhibited age-dependent decline and may contribute to ovarian failure and reproductive aging under physiological or pathological conditions.

## DISCUSSION

DNA damage in oocytes can cause infertility, abortion, and birth defects, leading to reproductive failure. Among all types of DNA damage, DSBs are the most deleterious and can substantially impair genome integrity. Experimental and clinical studies on mice and humans have revealed that intact DNA DSB repair in oocytes is necessary to preserve oocyte quantity and quality. Decline in DNA DSB repair has a causal relationship with ovarian failure, menopause, and infertility (Oktay et al., 2014, 2015; Titus et al., 2013; Xu et al., 2015). Genome-wide association studies on menopause timing have also highlighted the strong association of early menopause with DNA damage response pathways where many genes are involved in DNA repair, particularly the HR pathway (e.g., BRCA1 pathway) and checkpoint response (cell cycle response and apoptosis) (Day et al., 2015; Perry et al., 2013). Thus, investigating how oocytes respond to DNA DSBs and what molecules are involved in HR-mediated DSB repair will shed light on oocyte and ovarian aging. In this study, we presented the following lines of evidence to support the notion that OOEP may participate in HR-mediated DNA DSB repair by regulating the ATM-RAD51 axis in oocytes. Firstly, after etoposide treatment, γ-H_2_AX foci, which monitor DNA DSBs, were gradually resolved in wild-type oocytes but persisted in *Ooep* null oocytes, although this defect was rescued by re-expression of OOEP. Secondly, wild-type oocytes evoked the activation of ATM, a central coordinator in DNA DSB repair processes, in response to exogenous DNA damage. However, *Ooep*-/- oocytes failed to efficiently ignite ATM activation and this defect could be rescued by the re-introduction of OOEP proteins into mutant oocytes. Finally, RAD51, a recombinase essential for HR-mediated DNA DSB repair, was recruited to damage sites and formed foci in wild-type oocytes in response to etoposide treatment. In contrast, fewer RAD51 proteins were recruited to and formed foci at DNA DSBs in *Ooep*-/- oocytes, although this defect could also be rescued by exogenous OOEP.

Due to the compromised DNA damage repair, *Ooep*-/- oocytes displayed an accumulation of endogenous DNA damage, as determined by the comet assay, an unambiguous method that measures the extent of DNA damage on a single cell basis. As DNA damage can induce atresia of oocytes in primordial and primary follicles through the p63-mediated pathway (Gonfloni et al., 2009; Suh et al., 2006), we proposed that the accumulation of endogenous DNA damage in *Ooep*-/- oocytes may drive the atresia of oocytes and decrease the oocyte number in primordial and primary follicles under physiological conditions. Indeed, in young adult mice (16-week old), a mild but statistically significant decrease in oocyte quantity within the primordial and primary follicles was detected in the *Ooep*-/- ovaries compared to the wild-type counterparts. Oocytes in primordial and primary follicles from *Ooep*-/- females also displayed higher sensitivity to exogenous DNA insults and were prone to apoptosis. These observations suggest that OOEP is necessary for the preservation of oocyte quantity in normal and DNA-damaged conditions. OOEP depletion not only impairs the survival of oocytes within primordial and primary follicles, but also affects meiotic maturation and early embryonic development. Compared with wild-type oocytes, *Ooep*-/- GV oocytes exhibited delays in meiosis resumption as well as progression to the metaphase II stage. Moreover, embryos derived from *Ooep*-/- oocytes were arrested at the 2-cell stage (Li et al., 2008). This developmental arrest could be due to the DNA damage-induced cell cycle checkpoint (Xu et al., 2015). Thus, OOEP is essential for preserving oocyte quality. Interestingly, the mRNA expression of *Ooep* in mouse oocytes was reduced with advanced maternal age. Several key HR pathway genes, including *Brca1*, *Mre11*, *Atm*, and *Rad51*, also display age-dependent expression decrease in both human and mouse oocytes (Day et al., 2015; Oktay et al., 2015; Titus et al., 2013). Therefore, the coordinated decrease in the expression of *Ooep* and other canonical HR genes contributes to ovarian failure and reproductive aging under physiological and pathological conditions. OOEP is conservatively expressed in human oocytes (Zhu et al., 2015), implying that it may play similar roles in repairing DNA DSBs via the HR pathway in human oocytes.

OOEP proteins are distributed in cytoplasm and localized at the subcortex of mouse and human oocytes (Li et al., 2008; Zhu et al., 2015). OOEP was also excluded from the DNA DSB sites in oocytes treated with etoposide (data not shown). Thus, the functions of OOEP in regulating HR-mediated DNA DSB repair are likely indirect. OOEP is predicted to contain an atypical hnRNP K homology (KH) domain implicated in RNA binding (Wang et al., 2014). RNA binding proteins have been recognized as crucial players in the HR repair pathway (Adamson et al., 2012). The involvement of RBPs in regulating ovarian aging has also been reported (Day et al., 2015). For instance, RNA binding protein FMRP encoded by fragile X mental retardation (*Fmr1*) gene plays an essential role in preserving ovarian function in mice and humans (Ascano et al., 2012; Lu et al., 2012). In the future, elucidating the molecular mechanism of OOEP is warranted to better understand the function of RBPs in preserving the genome integrity of germ cells and early embryos.

## CONCLUSIONS

To the best of our knowledge, this work is the first to provide evidence that maternal OOEP may participate in HR-mediated DNA DSB repair by regulating the ATM-RAD51 axis in oocytes. Thus, OOEP may participate in the regulation of genome stability in oocytes and contribute to ovarian failure and reproductive aging under physiological or pathological conditions.
